# Multicentric study of epidemiological and clinical characteristics of persons injured in motor vehicle accidents in Medellín, Colombia, 2009-2010

**Published:** 2013-06-30

**Authors:** Luz Helena Lugo, Héctor Iván García, Blanca Cecilia Cano, Juan Carlos Arango, Olga Lucia Alcaraz

**Affiliations:** a Grupo de Rehabilitacion en Salud, Universidad de Antioquia E-mail: luzh.lugo@gmail.com; b Grupo Académico de Epidemiologia Clínica, Universidad de Antioquia. E-mail: higarcia@quimbaya.udea.edu.co; c Grupo de Rehabilitación en Salud E-mail: blancaceciliacano@gmail.com; d University of Deusto, Bilbao, España. E-mail: jcarango@deusto.es

**Keywords:** Traffic accidents, injury severity, New Injury Severity Score (NISS), quality of life, disability, WHODAS-II, SF-36

## Abstract

**Introduction:**

Traffic accidents (TA) cause 1.23 million deaths each year worldwide while between 20 and 50 million persons are injured each year. In 2011 in Medellin, Colombia, there were 307 traffic deaths and 23.835 injured with 411 accidents for each 10.000 vehicles.

**Objective:**

The purpose of the study was to describe the epidemiologic and clinical characteristics, as well as the quality of life and disability outcomes for those injured in traffic accidents in Medellin.

**Methods:**

This prospective, descriptive, cross-sectional study collected data from 834 patients that were classified with the New Injury Severity Score (NISS) , the WHO-DAS-II (Disability Assessment) Scale and the SF-36 Health Survey.

**Results:**

Three-fourths (75.8%) of the patients were male. Eighty-one percent (81.0%) of patients were involved in motorcycle accidents, with 45.6% suffering moderate trauma, and 32.6% experiencing severe trauma. Of the patients with severe trauma, 8.5% were not wearing helmets. Half of the sample (49.7%) injured their extremities. The WHODAS-II domains most affected were: Activities outside the home (62.0%), Housework (54.3%) and Moving in one's environment (45.2%). Quality of life areas affected were: Physical role (20.3%), Body pain (37.3%), Emotional role (44.1%), Physical functioning (52.6%).

**Conclusions:**

Patients with more severe injuries had higher levels of disability and a worse quality of life. Motorcycles made up a large proportion of traffic accidents in this city and mitigation strategies to reduce this public health problem should particularly focus on this high-risk group.

## Abstract (es)

**Antecedentes:**

Los accidentes de tránsito (AT) causan 1.23 millones de muertes en el mundo, y entre 20 y 50 millones sufren lesiones no fatales. En Medellín (Colombia) en el 2011 hubo 307 muertos en AT y 23.835 heridos, con 411 accidentes por cada 10.000 vehículos.

**Objetivo:**

Describir las características epidemiológicas, clínicas, de calidad de vida y discapacidad de los pacientes luego de un AT en Medellín.

**Métodos:**

Estudio descriptivo trasversal prospectivo del estado inicial de una cohorte de 834 pacientes, clasificados de acuerdo a la gravedad de trauma (NISS) y las escalas WHO-DAS II y SF-36.

**Resultados:**

En el 81% de los AT estuvieron involucradas motos, con lesiones moderadas en el 45.6 %, y graves en el 32.6% de ellos. No utilizaban casco el 8.5% de los pacientes con NISS grave. En el 49.7% se afectaron los miembros inferiores. Los dominios del WHO-DAS II más afectados fueron: actividades fuera del hogar (62.0%) actividades domésticas (54.3%) y moverse en el entorno (45.2%). La calidad de vida comprometió los dominios de desempeño físico (20.3%), dolor corporal (37.3%), desempeño emocional (44.1%) y funcionamiento físico (52.6%).

**Conclusiones:**

Los pacientes con lesiones más graves tuvieron mayor compromiso en la discapacidad y en la calidad de vida. Esta carga de enfermedad la producen principalmente los AT en los que están involucradas las motos.

## Introduction

The traffic injuries (TI) are a regional, national and global public health problem. According to the World Health Organization (WHO), more than 1.23 million persons die each year on the World's roads and between 20 and 50 million suffer non-fatal injuries. Although low and middle income countries have less than half of the World's vehicles, they produce over 90% of deaths related to TI. Forty-six percent (46%) of people who die from traffic accidents worldwide are vulnerable public road users, i.e. pedestrians, bicyclists and motorcyclists. WHO predicts that by 2030 trauma caused by traffic accidents which currently is the ninth leading cause of death, will occupy the fifth position, and in the absence of preventive measures, by 2020 it will cause 1.9 million deaths in the world each year^1^.

In Medellin, Colombia there were 307 deaths from traffic accidents and 23,835 injuries, with 411 accidents per 10,000 vehicles in 2011. Public transport and motorcycles accounted for 77% of the vehicles involved; motorcyclists, drivers and passengers accounted for 65% of those injured in accidents, and pedestrians accounted for 21% of the total^2^.

Trauma from traffic accidents was a major cause of disability in people of working age, with a greater involvement by men. These injuries have a negative impact on functioning, limitations to activity and restrictions on participation, as well as on the quality of life (QOL), emotional state, the ability, returning to work, and education^3^ ^,^ ^4^.

The consequences of trauma have been related to the severity of injuries with the most serious having worse functional consequences^5^ ^,^ ^6^. Soberg *et al*. found that six weeks after returning home after a traffic accident, patient functionality, as evaluated by the World Health Organization Disability Assessment Schedule II (WHO-DAS II) showed a low score (40.8) and that the general health and social functioning dimensions of the SF-36 health survey were low, 62 & 57 respectively^7^. In ESPARR cohort study, it was reported that six months after the trauma only 31.9% of patients believed that their health was as good as before the accident^8^.

This article describes the consequences for functioning, disability, and quality of life according to the severity of injuries among people who were involved in a traffic accidents in the city of Medellin, Colombia that formed the cohort group for a research study entitled, "Factors Related to Disability and Quality of Life in a group of persons injured in traffic accidents in the city of Medellin, 2009-2011".

## Materials and Methods

### Participants and design

This is a descriptive study of the initial evaluation of a cohort of patients taken from nine hospitals in Medellin. Eight hundred thirty four (834) patients between the ages of 16 and 60 years admitted to emergency services following a traffic accident in the city and its metropolitan area between March 2009 and December 2010 who resided in the same area agreed to participate in the research and signed an informed consent form were included.

The sample size of the group was calculated by taking the severity of the injury into account as a risk factor for disability, and with a ratio of severe and moderate to minor injuries of patients of 3:1, a 30% risk for severe and moderate patients, an 18.4% risk for those in the unexposed group, a difference in risk of 11.6 % at the 95% confidence level, a power of 80%, which yielded a sample size of 596 patients.

The calculated sample for the study was distributed among approved healthcare provider hospitals (i.e. IPS) according to the historical records for emergency care available in the city´s 123 system in 2008. They are as follows: Leo XIII University Hospital (31.2%), General Hospital of Medellin (23.5%), Clinic of the Americas (14.3%), Pablo Tobon Uribe Hospital (8.6%), Conquistador Clinic (7.9%), Bolivarian University Hospital (5.4%), San Vicente Foundation University Hospital (4.9%) and Soma Clinic (4.1 %). Patients were proportionally taken from minor, moderate and severe categories for each institution listed.

The cohort was consecutively assembled from the hospital lists by reviewing admission records for emergency services, and once the inclusion criteria were met, the patient was interviewed either on an outpatient basis if not hospitalized or at a place in the hospital or health service by appointment after being discharged if it was not possible to do so during hospitalization. Patients with traumatic brain injury (TBI) were interviewed after regaining consciousness and patients with cognitive limitations were not included. All interviews were conducted by healthcare professionals (psychologists, physiotherapists, social workers and doctors) or by medical students previously trained by the researchers. The study was approved by the Ethics Committee of the Research Office of the University of Antioquia and complied with the Ministry of Health of Colombia standards of Resolution 8430 (1993)

### Patient evaluation

Socio-demographic variables were collected including the characteristics of the accident, protective measures utilized, the nature of care, clinics (level of severity of the injury, bodily region affected, pain, functional impairment) and quality of life.

The severity of injury was based on the AISS (Abbreviated Injury Severity Scale) that ranks injuries from 1-6 with 1 minor, 2 moderate, 3 serious, 4 severe, 5 critical and 6 maximum (fatal injury). To assign the final score the NISS (New Injury Severity Score) was used, which is the sum of the square of the AIS score for each of the most serious injuries^9^. The values obtained were categorized as recommended by Soberg *et al*.^7^ which are: minor (NISS <4), moderate (NISS of 4-15) and severe (NISS > 15). Pain was measured by means of the Visual Analog Scale (VAS) from 0 "no pain" to 100 "maximum pain".

The WHO-DAS II scale is a sel-administered instrument to evaluate functional status in six life domains on a six-point ordinal scale from 1 "no difficulty for an activity" to 6 "extreme difficulty or unable to do". The domains are: Cognition (communication and understanding), Mobility (ability to move around the environment), Self-care (S), Getting Along (interactions with others, Life Activities (daily living activities at home and work) and Participation (community participation). A score for each domain was obtained and a summary value on a range of 0-100, from best to worst^10^.

QoL was assessed with the SF-36 Short Form Health Survey which is a generic scale validated in Colombia^11^ with 36 items in 8 domains: physical function (PF), physical role (RP), bodily pain (BP), general health (GH), vitality (VT), social functioning (SF), emotional role (RE) and mental health (MH). The scores obtained were placed on a scale of 0-100, where the higher the score the better the perception of the quality of life

### Statistical analysis

According to the type of variable summary statistics were used, including the mean and standard deviation (sd) or frequency distributions for characteristics of time, place and vehicle involved, demographics, clinical care, social security, accessibility to health services, disability and QoL. All these characteristics were compared according to the severity of the injury and the differences of patients with minor, moderate and severe injuries were evaluated using the Chi2 test or the more exact Fisher´s test for qualitative variables, for quantitative variables the Student's t-test was used if the variables were normally distributed (evaluated with the Kolmogorov Smirnov test) or the U of Mann Whitney if they did not. We considered a *p-value* <0.05 as significant.

## Results

### Demographic characteristics.

Eight hundred thirty-four (834) patients participated, 25.8% with minor injuries, 42.4% with moderate and 31.8% with severe injuries. Those with minor injuries were interviewed on an average of 15.9 days after the traffic accident (SD= 8.2), the second moderate group were interviewed on average 13.2 days afterward (SD= 12.2) days and those with severe injuries were interviewed an average of 11.2 days (SD= 12.1) days afterwards.Patients with moderate and severe injuries were younger, with a higher proportion of men mostly living alone and predominately receiving subsidized health care. There were no differences found between those with minor injuries versus those with moderate to severe injury concerning socioeconomic status, marital status, level of education and type of accident (*p*> .05). The demographic and social characteristics of the participants according to the severity of the injury are shown in Table 1.

**Table 1:**
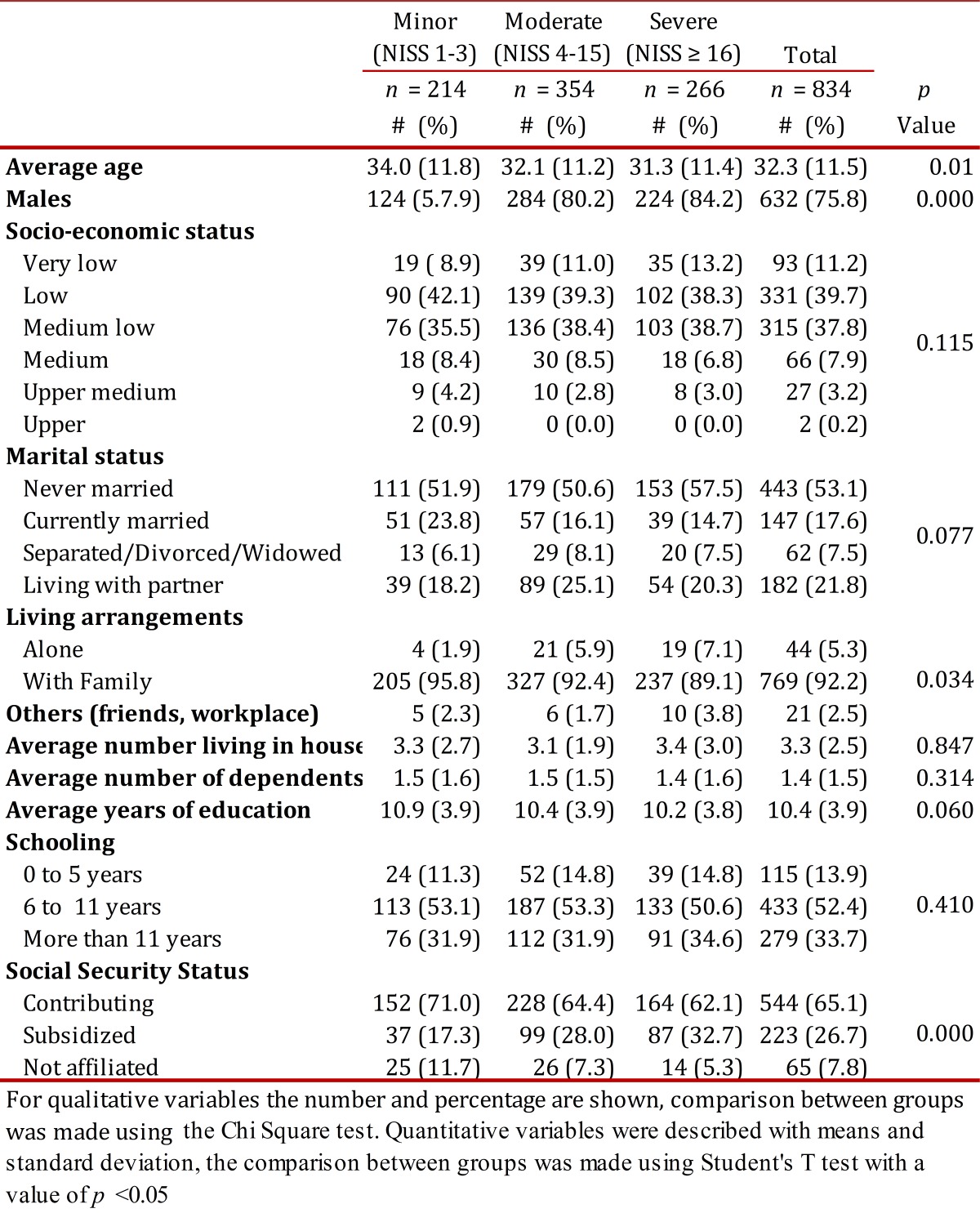
Social and demographic characteristics of the population according to the seriousness of the injuries

Almost seventy-six percent of patient (75.9%) were working, 9.1% students, 3.4% housewives, 10.8% unemployed (a third due to the traffic accident) and the remainder were retired. There were no differences found for injury severity level based on the type of occupation noted. The 50.4% of patients lived in their own home and 39% were renting or leasing. Only 0.2% of the sample lacked compulsory accident insurance (SOAT) and 2.3% did not know this information.

### Accident characteristics and initial care.

It was found that 27.5% of patients had had a previous traffic accident, but only 4.0% reported a prior disability. 51.3% of traffic accidents occurred between Friday and Sunday; 96.6% of them happened in the urban area and the largest number occurred in the center of the city. Eighty-one percent (81%) of traffic accidents involved motorcycles and 5.8% of persons with minor injuries were not using a helmet, while 7.2% of those with moderate injuries lacked a helmet and 8.5% of those severely injured. The proportion of patients with moderate to severe injuries as compared to those with minor injuries was greatest for motorcycle injuries (*p*= 0.001). of the 11.3% of traffic accidents involving cars, 71.4% of those with minor injuries were wearing seat belts; 83.3% of those with moderate injuries used seatbelts and 66.7% of those with severe injuries. Of the patients, 17.6% were under the influence of alcohol and, among those with severe injuries, this percentage rose to 22.7%; only 2.7% reported being under the influence of psychoactive substances. The seriously injured were taken on average to a hospital within 70.6 minutes (SD= 149), moderate injuries in 105 minutes (SD= 351) and minor injuries took an average of 186 minutes (SD= 672). Moderate and severely injured patients when compared with those with minor injuries were mainly taken in an ambulance or by firefighters. Tables 2 and 3 show the characteristics of traffic accidents with pre-hospital and hospital care.

**Table 2.**
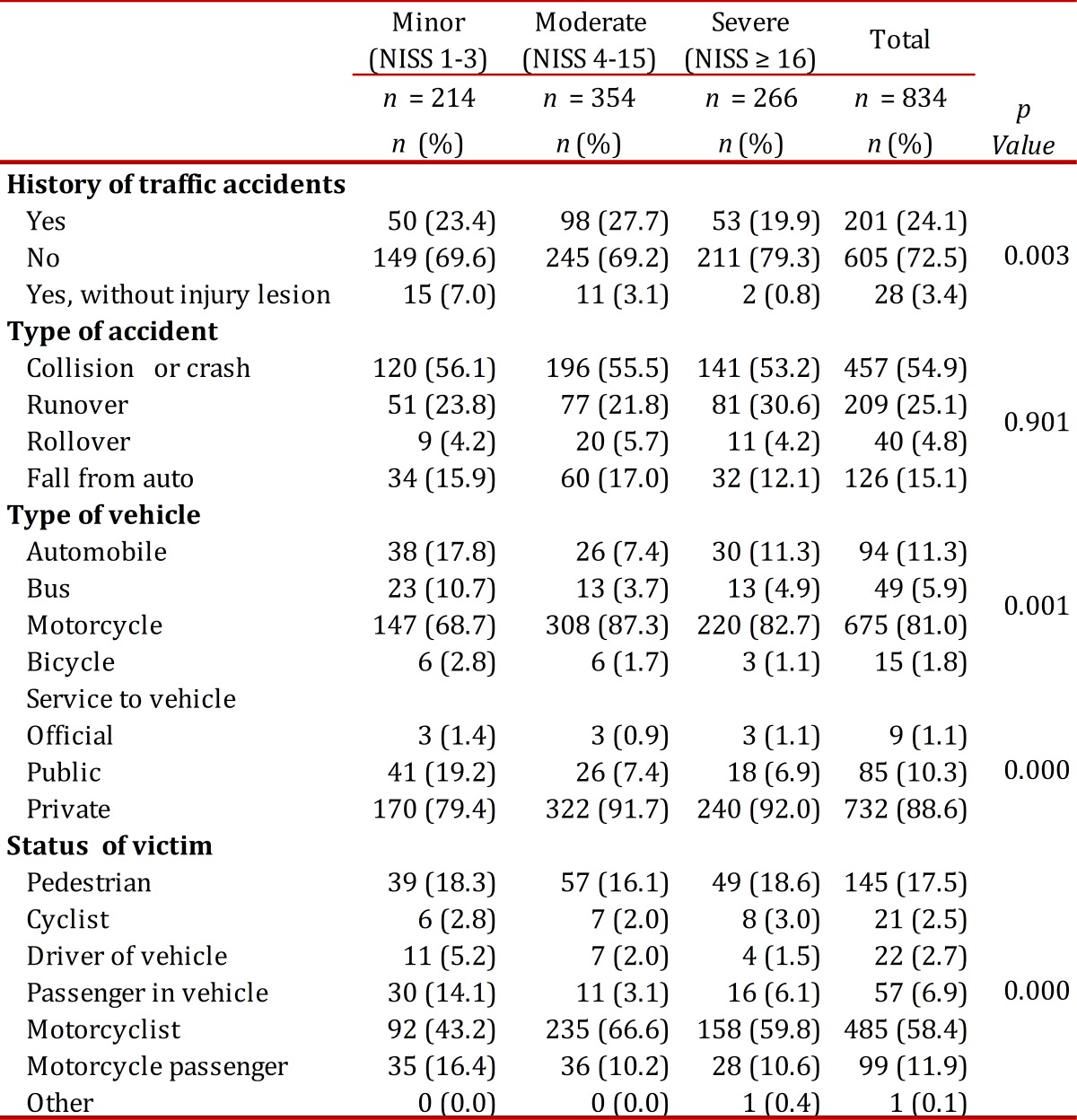
Accident characteristics according to severity of injuries For qualitative variables the number and percentage are shown, comparison between groups was made using the Chi2 test

**Table 3.**
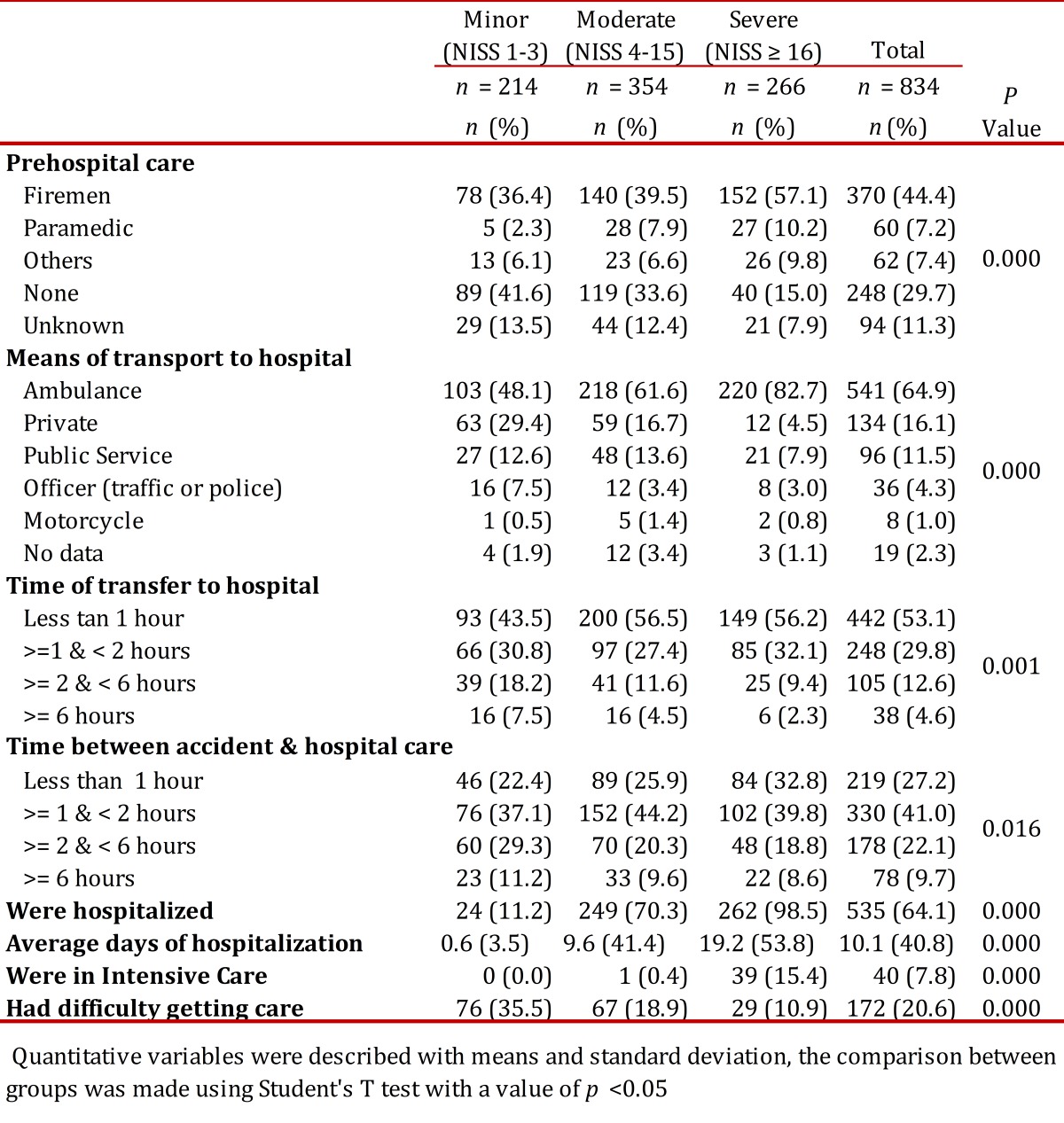
Characteristics of initial medical care according to the seriousness of the injury For qualitative variables the number and percentage are shown, comparison between groups was made using the Chi Square test.

### Clinical characteristics.

**Figure 1:**
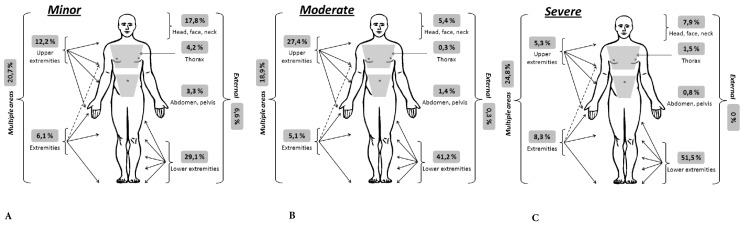
A,B,C: Compromise of bodily regions according to seriousness of injury.

The bodily regions most affected were the lower limbs in 49.7% of cases, the upper limbs in 23.8% and the face in 13.8% (Fig.1). Fifteen point three percent of patients had an ECT of those 128 who had a ECT, 31.3% had an NISS <4, 32.8%f those had a NISS between 4 and 15 while 35.9% had a NISS> 15. In terms of pain measured by the VAS it averaged 4.5 cm (SD= 3.0) in patients with minor NISS, 4.8 (SD= 2.8) in NISS moderate patients and 5.0 (SD= 2.8) with severe NISS. 51.2% of patients required initial medical disability, with the average number of days of disability of 20.5 (SD= 14.4) for minor cases, 36.6 (SD= 23.8) for moderate cases, and 30.9 (SD= 24.8) for the severe.

### Disability and quality of life.

The most affected domains from the WHODAS II were the CM, daily living domestic activities and daily activities outside the home for the three groups, with some differences according to the severity of the injury. Severe cases had an initial average of 62.1 (SD= 36) in domestic activities a 65 (SD= 36.3) and outside the home a 67.4 (SD= 35.2), and PC 48.2 (SD= 20.2).

When comparing disability among patients with moderate and severe injuries with minor ones there was greater compromise in the first group (*p* <0.05) in all domains, except for CC and RP, the latter two domains were the least compromised among the three groups (Table 4).

**Table 4.**
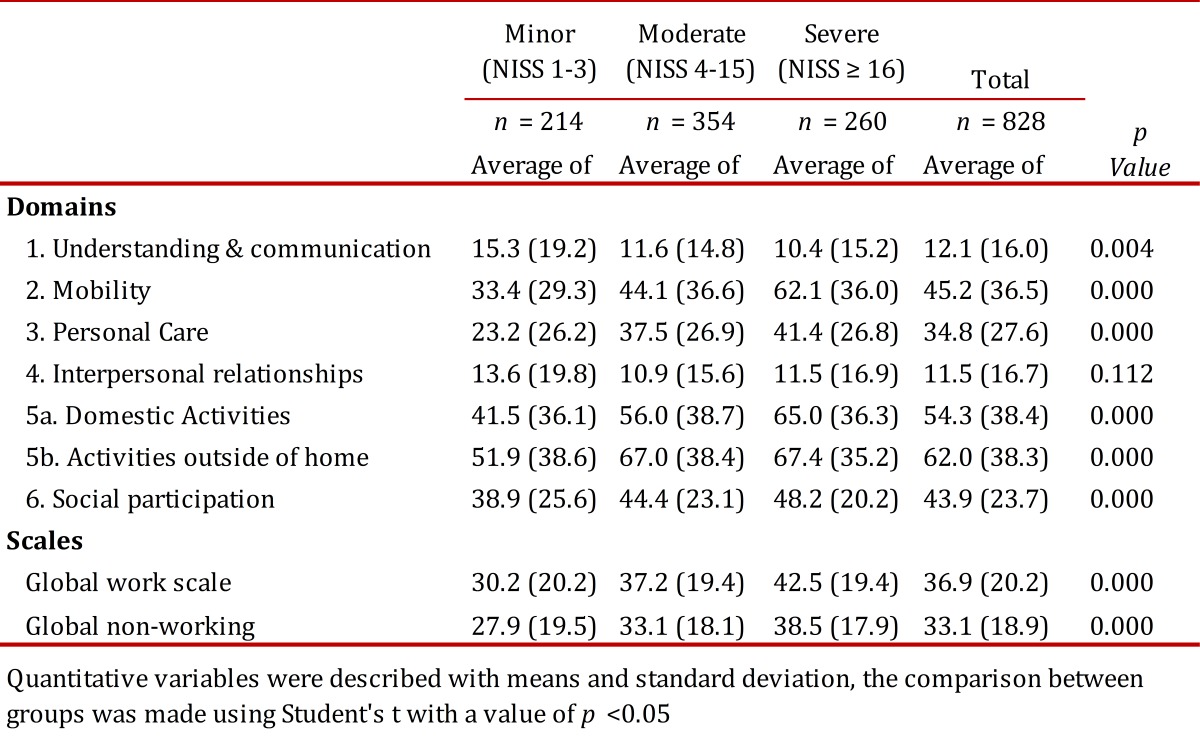
Disability as measured by WHODASII according to the seriousness of injury

In QoL the DF dimension was the most compromised in all groups, the FF had very low levels in severely injured patients of 12.5 (SD= 23.5) and 29.1 (sd = 30) score in moderately injured patients; the DC was low with 34 (SD= 24.6) in the three groups and the DE was the fourth category in all groups. 

**Figure 2.**
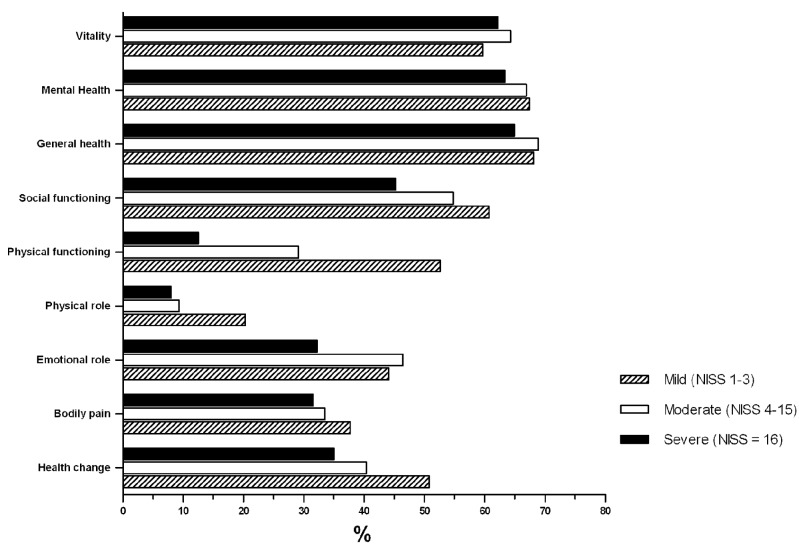
Quality of life, SF-36 according to seriousness of injury, p <0.05 in the domains of Health Change, Bodily Pain, Physical Role, Physical Function, Social Function, and Vitality. p> 0.05 in the domains of Emotional Role, General Health and Mental Health. We used Student's t test to compare the Minor versus Moderate + Severe.

There were differences in the CS, DC, DF, FF, FS and VT dimensions when comparing severe and moderate patients with minor (*p* <0.05). The less compromised dimensions were SG, SM and VT, although none had an average greater than 70 (Fig. 2).

## Discussion

Colombia, a country of medium per capita income, has is a traffic accident rate of 411 per 10,000 vehicles, which is higher than that occurring in Africa with an average rate of 68 per 10,000 vehicles. Traffic accidents in developing countries are more than double that of developed countries with 13.4/100,000 and 32.2/100,000 persons in Europe and Africa respectively^12^.

Traffic accidents occur among young persons as shown in this study and various publications, the highest average was 35.3 years and men were involved in more than 70% of cases^5^ ^,^ ^7^ ^,^ ^13^. In a study in Kenya, the average age of victims was 32.4 years, 75% were between 20 and 49 years of age, 12% were over 50 years old and most were men (73%) 12. In our cohort, most patients lived with their family even though only 39.4% lived with their partner which differs from Soberg´s *et al*. study in which they account for 55% 7. In a French study with 1168 persons, 65% were employed 8 in our study 75.9% were employed, with 27.2% having independent work.The results of this study show that traffic accidents more often occur on weekends, 19.5% on the Saturday, as has been described in several studies, while fatal accidents occur more often at night^14^. Aslam *et al*. showed in Pakistan that accidents occurred most often on Saturday (31.0%)^15^. In relation to the characteristics of traffic accidents we observed that collision was the most frequent type with a percentage of 54.9%, which is similar to other studies^16^. Accidents involving a motorcycle presented a greater frequency of severe injury, 2.3 times greater 13. The ESPARR cohort data showed that less serious injuries tended to be with 4-wheel vehicles (51.7%) and the most severely injured patients were users of two-wheeled vehicles in 37.5% of cases^8^.

In this cohort, 81% of traffic accidents involved motorcycles, which is thought due to increasing numbers in the city, the lack of effective educational programs and regulations and non-compliance with traffic laws.

The ESPARR cohort data showed that less serious injuries tended to occur with four-wheel vehicles (51.7%), and the most serious injuries occurred to users of two-wheeled vehicles (37.5%). In a French study, motorcycle riders were involved in 30.2% of traffic accidents 8, while in India the percentage was 59.7%^17^. Pedestrians composed 17.5% of patients, with no differences noted by NISS category and it was thought possible that this figure would have been higher if persons over 60 years of age were included. In the ESPARR cohort the proportion of pedestrians was greater in the most severely injured group^8^.

The number of motorcycles circulating in the country and in Medellin has increased in recent years; according to data from the registry of active vehicles, in 2011 the total number of motorcycles registered in the city was 26,280. 

The road fleet has increased in other Latin American countries, such as Brazil^18^, as well as in Asia and Europe. In Brazil there are more than 14 million motorcycles in circulation, 25% of the national fleet, which has also increased the number of motorcycle accidents^18^. In India in 2004, motorcycle users represented 70% of total number of registered vehicles^17^. In Thailand, 80% of accidents involve motorcycles and in Malaysia more than 50% of those who die in traffic accidents are motorcyclists. In these two countries, two-wheeled vehicles account for over 50% of registered vehicles compared to Kenya where they only account for 7.7% of all licensed vehicles^12^.

Among possible causes for the increased number of circulating motorcyles are: greater ease of mobility, decreased travel time, a more economical means of travel, mass transit deficiencies and for some people it is their means of working. 

Helmet usage was high in this study, as has been found in other studies^8^ but different from Fitzharris *et al*. study done in India where only 19.6% used a helmet and also differing from the study by Hatamabadi *et al*. in Iran where only 26.5% of motorcyclists wore a helmet^19^.

In a study conducted at a single hospital in Medellin by Garcia *et al*. it was observed that in traffic accidents the driver's recklessness was the most frequent reason (38.8%) given for it occurring, followed by loss of control or falls from a moving vehicle (22.3%), recklessly crossing the road (5.7%), the violation of traffic rules (5.6%) and mechanical failure of the vehicle (4.6%). In summary, over half were attributed to the driver and a fifth to multiple and unspecified factors^20^.

Alcohol is the drug most often associated with all kinds of traffic accidents, and as other studies have reported motorcycle drivers are the most frequent consumers of alcohol in both fatal and non-fatal accidents^21^ ^,^ ^22^. It was observed in this cohort that patients with a severe NISS were those with a higher percentage of reported alcohol consumption (22.7%).

Lower limb injuries were the most common type of injury, which is similar to other studies, such as by Lin^22^ and ESPARR, where they accounted for 67.8% of all injuries^8^, and similarly in Turkey where it was reported that body area most affected were the lower extremities (62.9%)^23^.

The results of the WHO-DAS II assessment showed that trauma patients appear to have greater disability than that reported for other diseases, such as Ankylosing Spondylitis, rheumatoid arthritis or general chronic musculoskeletal conditions^24^. The Soberg study conducted with 107 patients with ages between 18 and 67 years with severe injuries (NISS ≥ 16) showed after an average of 6 weeks a WHO-DAS II total of 40.7 (SD= 15.9). The most compromised domains in our study were activities outside the home, 67.4 (SD= 35.2) and domestic activities, 65.0 (SD= 36.3). In the study by Soberg the most compromised domains were Life Activities, which comprise the two previous categories noted with a median of 50.0 (IQR: 35.0 - 80.0). The next most compromised areas were Mobility with a score of 62.1 (SD= 36.0) versus. 37.5 (IQR: 12.5-62.5), Social Participation 48.2 (SD= 20.2) versus 45.8 (IQR: 37.5-58.3), Self-Care 41.4 (SD= 26.8) versus 20.0 (IQR 0.0-30.0). In both studies, compromised functioning, restriction of activities and participation constraints are very important; however, it was greater in this study probably due to the initial assessment being completed during the first two weeks. The domains less compromised in both studies were understanding and communication, i.e. Cognition, and interpersonal relationships, i.e. Getting Along^25^.

Quality of life was more compromised in our study than shown in Soberg´s 7. For example, physical functioning was 8 (SD= 25.1) versus 45.6 (SD= 28.8), bodily pain was 31.5 (SD= 23.7) versus 51.1 (SD= 28), and the emotional role was 32.2 (SD= 42.6) versus 51.7 (SD= 45.1). This difference in scores is likely due the questionnaire being completed during the more acute stage immediately following the traffic accident for a great percentage of the patients evaluated in our cohort. A greater compromise was observed in the Soberg *et al* study for the domains of physical function and vitality; the physical functioning in this study was 12.5 (SD= 23.5) verus. 9.47 (SD= 20.7), and Vitality was 62.1 (SD= 19.1) versus 46.8 (SD= 20.9).

Some limitations existed in this study especially concerning gathering information from ambulatory patients, some of whom did not want to participate because they were not financially compensated, or were afraid of giving information that might be compromising. Cultural awareness is still lacking in our country with regard to the importance of scientific research. In some hospitals there were difficulties in obtaining all the information about the time of care following the traffic accident and other data concerning patient care. Other limitations of the study were the exclusion of patients over 60 years of age, and that there is no recognized trauma classification system in our country.Although traffic accidents are a major cause of death and disability worldwide and several risk factors have been identified for their occurrence, only 15% of countries have comprehensive laws related to the five major risk factors: excessive speed, driving under the influence of alcohol, mandatory use of helmets by motorcyclists, usage of seat belts and safety seats for children. In this country the number of motorcycles and accidents related to them continue to increase, driver educational programs and appropriate vehicle regulations for private cars and motorbikes are still lacking.
